# Isotretinoin-induced Intracranial Hypertension Presenting as Unilateral Pulsatile Tinnitus

**DOI:** 10.1097/ONO.0000000000000086

**Published:** 2026-03-18

**Authors:** Kaeden L. McClintock, Kathryn Wie, Daniel H. Coelho

**Affiliations:** Department of Otolaryngology, VCU School of Medicine, Richmond, VA.

**Keywords:** Intracranial hypertension, Isotretinoin, Pulsatile tinnitus

## Abstract

**Background:**

Pulsatile tinnitus (PT), a challenging condition for patients and clinicians alike, is often linked to vascular, neoplastic, and systemic disorders. The most common cause is idiopathic intracranial hypertension (IIH). However, physicians must recognize that not all intracranial hypertension (IH) is idiopathic and can be due to secondary causes, including cerebral venous anomalies, medications, and systemic medical conditions.

**Objective:**

To highlight the importance of this entity, we report a case of drug-induced IH (DIIH), caused by isotretinoin (ISO), that presented with persistent unilateral PT.

**Methods:**

Informed consent for the publication of clinical information was obtained from the patient.

**Results:**

A 30-year-old female developed unilateral PT and blurred vision shortly after initiating ISO for acne. She was diagnosed with IH, and despite ISO cessation, acetazolamide escalation, and multiple therapeutic lumbar punctures, her PT persists 1 year later. Neuroimaging revealed a high-riding jugular bulb with diverticulum and possible sigmoid sinus dehiscence, likely contributing to the persistence of her symptoms following resolution of her headaches and visual changes.

**Conclusion:**

While DIIH typically resolves after discontinuation of the offending agent, this case demonstrates a unique presentation of ISO-associated DIIH manifesting as unilateral PT that persisted despite medical therapy and drug cessation. This highlights the importance of recognizing DIIH as a potential cause of PT, as well as evaluating coexisting anatomic variants and medical comorbidities, which are crucial for timely diagnosis and treatment.

Pulsatile tinnitus (PT), a rhythmic auditory sensation synchronous with a patient’s heartbeat, is a potentially debilitating condition that affects an estimated 3–5 million Americans, which can lead to substantial psychological distress and reduced quality of life (1). Because etiologies range from vascular to neoplastic and systemic disorders, diagnosis and treatment have historically been challenging (2–4). As imaging modalities have become more sophisticated, comprehensive algorithmic approaches to the diagnosis of PT have improved detection of the underlying etiology, with recent studies demonstrating diagnostic yield of greater than 90% (5–10).

One of the most common causes of PT is idiopathic intracranial hypertension (IIH), formerly known as pseudotumor cerebri (10). While classically presenting with a triad of headache, visual changes, and papilledema in an overweight female of childbearing age, IIH has recently expanded to include a broad array of clinical presentations including PT, the most frequently encountered otoneurological symptom (11–13). Importantly, although rare, not all intracranial hypertension (IH) is idiopathic and may be due to secondary causes such as cerebral venous anomalies, medications, or systemic medical conditions (14). Here, we report a unique case of persistent unilateral PT in a patient with drug-induced IH (DIIH) caused by isotretinoin (ISO).

## METHODS

Informed consent for the publication of clinical information was obtained from the patient.

## CASE PRESENTATION

A 30-year-old female with a body mass index of 29 kg/m^2^ noted right PT for approximately 6 months before presentation, for which she previously did not seek medical attention. After the onset of PT, she also experienced episodic headaches, cloudy vision, and blackouts, prompting evaluation in the emergency room. She was referred by her optometrist, who had noted optic nerve edema, and she was subsequently found to have bilateral papilledema. She denied any ocular pain, drainage, or discharge, diplopia, or focal numbness, tingling, or weakness. Her neurological exam was otherwise normal. A computed tomography scan of her head was unremarkable, and her lumbar puncture opening pressure (LPOP) was elevated at 25.5 cm H_2_O with normal cerebrospinal fluid (CSF) composition. Further questioning revealed that she began oral ISO (40 mg daily) 3 months before PT onset. She was diagnosed with IH, the ISO was stopped, and she was started on acetazolamide 250 mg twice daily.

Two months later, she was referred to our otolaryngology clinic for evaluation of persistent right unilateral PT despite moderately improved headache and visual symptoms. She reported her PT occurred spontaneously throughout the day without obvious triggers, and denied any associated hearing changes, ear pain or fullness, vertigo, or autophony. Physical exam was notable for a negative bruit, decreased PT on internal jugular vein compression, and tinnitus corresponding to arterial pulsations. Otoscopic examination and audiometry were normal. Noncontrast computed tomography of the temporal bone was notable for a large right jugular bulb with a small lateral diverticulum and a subcentimeter area of possible superior sigmoid sinus dehiscence without diverticulum. Carotid Doppler ultrasound demonstrated less than 50% stenosis of the internal carotid arteries and antegrade flow in the vertebral arteries. Magnetic resonance imaging of her head demonstrated no acute intracranial, orbital, or optic nerve abnormalities. Consequently, her PT was attributed to DIIH exacerbating flow abnormalities through her anomalous ipsilateral jugular bulb.

Given only moderate improvement of symptoms with persistence of PT, her acetazolamide regimen was increased to 250 mg thrice daily to maximize medical management. At a follow-up evaluation with her ophthalmologist 1 month later, 3 months since stopping ISO and starting acetazolamide, she reported complete resolution of her visual symptoms but endorsed continued PT and demonstrated persistent papilledema on fundoscopic examination. Two months later, her neurologist also observed another elevated LPOP of 28 cm H_2_O, and she reported no resolution of her PT after a therapeutic lumbar puncture. Her acetazolamide dose was then further increased to 1*g* daily, and she continues to endorse persistent PT at nearly 1 year since initiation of medical therapy and declined surgical sigmoid resurfacing with diverticulum reduction for symptomatic relief.

## DISCUSSION

The most common form of IH is IIH, defined by the Modified Dandy Criteria including symptoms of increased intracranial pressure (ICP; eg, progressive headache), signs of increased ICP (eg, papilledema, visual field defects, or abducens nerve palsy) with otherwise normal neurologic exam and neuroimaging, and elevated LPOP with normal CSF composition (12,13). Early diagnosis is critical, as timely intervention can prevent progressive, irreversible vision loss (15). Recent neuroimaging advances have expanded the recognized spectrum of IIH, including atypical presentations such as isolated PT (11,12).

Although IIH is strongly associated with obesity, secondary causes of IH including venous anomalies, medications, and systemic conditions, must also be considered (14). Obstructive sleep apnea is a particularly relevant comorbidity, with nearly 50% prevalence among IIH patients (16). Screening tools such as the STOP-Bang Questionnaire, Epworth Sleepiness Scale, or overnight polysomnography are readily available, and several studies have demonstrated improvement in IIH following obstructive sleep apnea treatment with continuous positive airway pressure or oropharyngeal surgery (17–20).

DIIH has been reported with numerous agents (Table 1) (21). Retinoids such as ISO are among the most common, thought to increase ICP by stimulating CSF production and impairing resorption (21,22). Symptoms usually resolve within days to weeks after discontinuation and initiation of acetazolamide to reduce ICP, though rare tetracycline-associated cases may require long-term therapy or surgical intervention (23). Most reports of ISO-associated DIIH describe resolution of symptoms, including PT, days-to-weeks after cessation of ISO (24–32). Only 1 case of ISO-associated DIIH reported persistence of symptoms (headache and bilateral vision loss; PT was not reported) despite cessation of ISO and tetracycline and long-term medical therapy, ultimately requiring bilateral nerve sheath decompression (33).

**TABLE 1.** Medications implicated in drug-induced IH

Application	Medication
Dermatologic	Vitamin A, Isotretinoin, Tretinoin, Minocycline, Tetracycline, Doxycycline, Spironolactone, Oxytetracycline
Endocrine	Human Growth Hormone, Corticosteroids, Leuprorelin, Triptorelin, Norplant, Nexplanon, Levonorgestrel, Medroxyprogesterone, Combined OCPs, Stanozolol, Danazol, Duraglutide, Desmopressin
Antimicrobial	Nalidixic Acid, Ciprofloxacin, Levofloxacin, Ofloxacin, Gentamicin/Penicillin
Antifungal	Ketoconazole
Rheumatologic	Cyclosporine, Sulfasalazine, Ustekinumab, Methotrexate, Mycophenolate, Budesonide
Antiarrhythmic	Amiodarone
Psychiatric	Lithium, Divalproic Acid, Mirtazapine, Risperidone, Fluvoxamine

IH indicates intracranial hypertension; OCPs, oral contraceptive pills.

PT is reported in 52%–60% of patients with IH, two-thirds of whom present bilaterally (34). Mechanistically, IH alters cerebral venous flow, producing turbulence within the transverse and sigmoid sinuses that transmits vascular pulsations through the temporal bone, amplifying them at the cochlea (35). Additionally, anatomical variants such as sigmoid sinus dehiscence or diverticulum are more prevalent in patients with IH and have been shown to increase PT risk (36–39). Reports of PT laterality in DIIH throughout published literature are limited. However, in a radiographic study of 170 patients with unilateral PT, ipsilateral anatomic abnormalities, including dehiscent sigmoid plate, high jugular bulb, sigmoid sinus diverticulum, and dehiscent jugular bulb, were identified in over 70% of cases (40). This supports the hypothesis that this patient’s unilateral PT may be attributed to DIIH exacerbating flow abnormalities through her anomalous ipsilateral jugular bulb. Furthermore, while reduction of ICP via lumbar puncture or medical therapy can decrease venous sinus flow velocity, a recent retrospective review demonstrated persistence of PT in most patients with IH despite resolution of papilledema, as was also observed in this patient’s case (41).

This case is unique as it represents the first report of ISO-associated DIIH presenting with unilateral PT that persisted after ISO cessation. The patient’s ipsilateral high-riding right jugular bulb with a diverticulum and possible sigmoid sinus dehiscence likely contributed to her unilateral PT in the setting of DIIH. This report adds to growing evidence that medications can cause IH and present with PT. As otolaryngologists increasingly encounter PT, it is essential to recognize that not all IH is idiopathic. Thorough medication review, evaluation of medical comorbidities, and algorithmic imaging approaches are critical to identify pharmacologic, physiologic, and anatomic contributors to PT.

## FUNDING SOURCES

None declared.

## CONFLICTS OF INTEREST

None declared.

## DATA AVAILABILITY STATEMENT

All relevant data is contained within the article: The original contributions presented in the study are included in the article/supplementary material, further inquiries can be directed to the corresponding author.

## References

[1] Amans M Mattay R Hills NK. More than just noise: association of pulsatile tinnitus with anxiety, depression, and reduction of quality of life. Interv Neuroradiol. 2023;31(3):381–385. 37069825 10.1177/15910199231168751 PMC12202887

[2] Sonmez G Basekim CC Ozturk E Gungor A Kizilkaya E. Imaging of pulsatile tinnitus: a review of 74 patients. Clin Imaging. 2007;31:102–108. 17320776 10.1016/j.clinimag.2006.12.024

[3] Lynch P Mitton T Killeen DE Kutz JW Newcomer M. Diagnosing pulsatile tinnitus: a review of 251 patients. Otol Neurotol. 2022;43:128–136. 34629443 10.1097/MAO.0000000000003370

[4] Waldvogel D Mattle HP Sturzenegger M Schroth G. Pulsatile tinnitus —a review of 84 patients. J Neurol. 1998;245:137–142. 9553842 10.1007/s004150050193

[5] Tao AJ Parikh NS Patsalides A. The role of noninvasive imaging in the diagnostic workup for pulsatile tinnitus. Neuroradiol J. 2022;35:220–225. 34459683 10.1177/19714009211036696 PMC9130612

[6] Mattox DE Hudgins P. Algorithm for evaluation of pulsatile tinnitus. Acta Otolaryngol. 2008;128:427–431. 18368578 10.1080/00016480701840106

[7] Narsinh KH Hui F Saloner D. Diagnostic approach to pulsatile tinnitus: a narrative review. JAMA Otolaryngol. 2022;148:476–483. 10.1001/jamaoto.2021.4470 35201283

[8] Grierson KE Bou-Haidar P Dumper J Fagan PA. The assessment of pulsatile tinnitus—a systematic review of underlying pathologies and modern diagnostic approaches. Aust J Otolaryngol. 2018;1.

[9] Duvvuri M Ali H Amans MR. Non-invasive imaging modalities for diagnosing pulsatile tinnitus: a comprehensive review and recommended imaging algorithm. J Neurointerventional Surg. 2025;17(9):916–924. 10.1136/jnis-2023-020949 PMC12290034 39488339

[10] Sismanis A Coelho D. Improving diagnostic yield in patients with pulsatile tinnitus: a ten-year analysis. Laryngoscope. 2025;135(3):1148–1154. 39440415 10.1002/lary.31838

[11] Biousse V Newman NJ. The expanding spectrum of idiopathic intracranial hypertension. Eye. 2023;37:2361–2364. 36509997 10.1038/s41433-022-02361-3 PMC10397341

[12] Chen BS Britton JOT. Expanding the clinical spectrum of idiopathic intracranial hypertension. Curr Opin Neurol. 2023;36:43–50. 36444979 10.1097/WCO.0000000000001131 PMC9835678

[13] Stevens SM Rizk HG Golnik K. Idiopathic intracranial hypertension: contemporary review and implications for the otolaryngologist. Laryngoscope. 2018;128:248–256. 28349571 10.1002/lary.26581

[14] Wang MTM Bhatti MT Danesh-Meyer HV. Idiopathic intracranial hypertension: pathophysiology, diagnosis and management. J Clin Neurosci. 2022;95:172–179. 34929642 10.1016/j.jocn.2021.11.029

[15] Ball AK Clarke CE. Idiopathic intracranial hypertension. Lancet Neurol. 2006;5:433–442. 16632314 10.1016/S1474-4422(06)70442-2

[16] Yiangou A Mitchell JL Nicholls M. Obstructive sleep apnoea in women with idiopathic intracranial hypertension: a sub-study of the idiopathic intracranial hypertension weight randomised controlled trial (IIH: WT). J Neurol. 2022;269:1945–1956. 34420064 10.1007/s00415-021-10700-9 PMC8940816

[17] Javaheri S Qureshi Z Golnik K. Resolution of papilledema associated with OSA treatment. J Clin Sleep Med. 2011;7:399–400. 21897778 10.5664/JCSM.1202 PMC3161773

[18] Kalyoussef E Brooks NO Quraishi H Turbin R Frohman L. Idiopathic intracranial hypertension in a child with obstructive sleep apnea cured by tonsillectomy/adenoidectomy. J Neuro Ophthalmol. 2013;33:413–414. 10.1097/WNO.0000000000000059 24121900

[19] Onder H Ergun O Kaygisiz M Daltaban IS. Total improvement after surgery for obstructive sleep apnea syndrome in a patient with concurrent malignant idiopathic intracranial hypertension. J Neurosurg. 2019;131:582–586. 30117763 10.3171/2018.3.JNS171663

[20] Purvin VA Kawasaki A Yee RD. Papilledema and obstructive sleep apnea syndrome. Arch Ophthalmol. 2000;118:1626–1630. 11115256 10.1001/archopht.118.12.1626

[21] Tan MG Worley B Kim WB Ten Hove M Beecker J. Drug-induced intracranial hypertension: a systematic review and critical assessment of drug-induced causes. Am J Clin Dermatol. 2020;21:163–172. 31741184 10.1007/s40257-019-00485-z

[22] Warner JEA Bernstein PS Yemelyanov A. Vitamin A in the cerebrospinal fluid of patients with and without idiopathic intracranial hypertension. Ann Neurol. 2002;52:647–650. 12402264 10.1002/ana.10377

[23] Angelette AL Rando LL Wadhwa RD. Tetracycline-, doxycycline-, minocycline-induced pseudotumor cerebri and esophageal perforation. Adv Ther. 2023;40:1366–1378. 36763302 10.1007/s12325-023-02435-y

[24] Reifenrath J Rupprecht C Gmeiner V Haslinger B. Intracranial hypertension after rosacea treatment with isotretinoin. Neurol Sci. 2023;44:4553–4556. 37646978 10.1007/s10072-023-07039-6 PMC10641047

[25] Rosende L Verea-Hernando MM de Andrés A. Hypoacusia in a patient treated by isotretinoin. Case Rep Med. 2011;2011:789143. 22162704 10.1155/2011/789143 PMC3227444

[26] Yaman M Albayram S Altintas A Yeni SN Karaagac N Islak C. A cerebellar demyelinating lesion following treatment of acne with isotretinoin. Clin Exp Dermatol. 2008;33:118–121. 17501969 10.1111/j.1365-2230.2007.02429.x

[27] Fraunfelder FT LaBraico JM Meyer SM. Adverse ocular reactions possibly associated with isotretinoin. Am J Ophthalmol. 1985;100:534–537. 2931989 10.1016/0002-9394(85)90676-2

[28] Fraunfelder FW Fraunfelder FT Corbett JJ. Isotretinoin-associated intracranial hypertension. Ophthalmology. 2004;111:1248–1250. 15177980 10.1016/j.ophtha.2003.09.044

[29] Flores V Willman J Willman M Prakash R Lucke-Wold B. A case of idiopathic intracranial hypertension and concomitant postural orthostatic tachycardia syndrome. Adv Case Stud. 2024;4:1–7.

[30] Vashisht KR Varma S Yeganegi M. Isotretinoin-associated intracranial hypertension: a concise report. JEADV Clin Pract. 2024;3:1627–1630.

[31] Spector RH Carlisle J. Pseudotumor cerebri caused by a synthetic vitamin A preparation. Neurology. 1984;34:1509–1511. 6238241 10.1212/wnl.34.11.1509

[32] Lombaert A Carton H. Benign intracranial hypertension due to A-hypervitaminosis in adults and adolescents. Eur Neurol. 1976;14:340–350. 133026 10.1159/000114758

[33] Lee AG. Pseudotumor cerebri after treatment with tetracycline and isotretinoin for acne. Cutis. 1995;55:165–168. 7634848

[34] Markey KA Mollan SP Jensen RH Sinclair AJ. Understanding idiopathic intracranial hypertension: mechanisms, management, and future directions. Lancet Neurol. 2016;15:78–91. 26700907 10.1016/S1474-4422(15)00298-7

[35] Haraldsson H Leach JR Kao EI. Reduced jet velocity in venous flow after CSF drainage: assessing hemodynamic causes of pulsatile tinnitus. AJNR Am J Neuroradiol. 2019;40:849–854. 31023664 10.3174/ajnr.A6043 PMC6613781

[36] Liu Z Dong C Wang X. Association between idiopathic intracranial hypertension and sigmoid sinus dehiscence/diverticulum with pulsatile tinnitus: a retrospective imaging study. Neuroradiology. 2015;57:747–753. 25845808 10.1007/s00234-015-1517-5

[37] Kline NL Angster K Archer E. Association of pulse synchronous tinnitus and sigmoid sinus wall abnormalities in patients with idiopathic intracranial hypertension. Am J Otolaryngol. 2020;41(6):102675. 32854043 10.1016/j.amjoto.2020.102675

[38] Zhao P Jiang C Lv H Zhao T Gong S Wang Z. Why does unilateral pulsatile tinnitus occur in patients with idiopathic intracranial hypertension? Neuroradiology. 2021;63:209–216. 32880675 10.1007/s00234-020-02541-6

[39] Xu S Ruan S Liu S Xu J Gong R. CTA/V detection of bilateral sigmoid sinus dehiscence and suspected idiopathic intracranial hypertension in unilateral pulsatile tinnitus. Neuroradiology. 2018;60:365–372. 29417173 10.1007/s00234-018-1987-3

[40] Dong C Zhao P-F Yang J-G Liu Z-H Wang Z-C. Incidence of vascular anomalies and variants associated with unilateral venous pulsatile tinnitus in 242 patients based on dual-phase contrast-enhanced computed tomography. Chin Med J (Engl). 2015;128:581–585. 25698187 10.4103/0366-6999.151648 PMC4834766

[41] Snowden A Van Stavern GP Stunkel L. Persistence of pulsatile tinnitus in patients with idiopathic intracranial hypertension following resolution of papilledema. J Neurol Sci. 2025;476:123608. 40695204 10.1016/j.jns.2025.123608

